# Harnessing CD8^+^ T Cells Under HIV Antiretroviral Therapy

**DOI:** 10.3389/fimmu.2019.00291

**Published:** 2019-02-26

**Authors:** Joanna A. Warren, Genevieve Clutton, Nilu Goonetilleke

**Affiliations:** ^1^Department of Microbiology and Immunology, University of North Carolina, Chapel Hill, NC, United States; ^2^UNC HIV Cure Center, University of North Carolina, Chapel Hill, NC, United States

**Keywords:** HIV, CD8 T cell, antiretroviral therapy (ART), HIV cure strategies, aging, immunosenescence, vaccines

## Abstract

Antiretroviral therapy (ART) has transformed HIV from a fatal disease to a chronic condition. In recent years there has been considerable interest in strategies to enable HIV-infected individuals to cease ART without viral rebound, either by purging all cells infected harboring replication-competent virus (HIV eradication), or by boosting immune responses to allow durable suppression of virus without rebound (HIV remission). Both of these approaches may need to harness HIV-specific CD8^+^ T cells to eliminate infected cells and/or prevent viral spread. In untreated infection, both HIV-specific and total CD8^+^ T cells are dysfunctional. Here, we review our current understanding of both global and HIV-specific CD8^+^ T cell immunity in HIV-infected individuals with durably suppressed viral load under ART, and its implications for HIV cure, eradication or remission. Overall, the literature indicates significant normalization of global T cell parameters, including CD4/8 ratio, activation status, and telomere length. Global characteristics of CD8^+^ T cells from HIV^+^ART^+^ individuals align more closely with those of HIV-seronegative individuals than of viremic HIV-infected individuals. However, markers of senescence remain elevated, leading to the hypothesis that immune aging is accelerated in HIV-infected individuals on ART. This phenomenon could have implications for attempts to prime *de novo*, or boost existing HIV-specific CD8^+^ T cell responses. A major challenge for both HIV cure and remission strategies is to elicit HIV-specific CD8^+^ T cell responses superior to that elicited by natural infection in terms of response kinetics, magnitude, breadth, viral suppressive capacity, and tissue localization. Addressing these issues will be critical to the success of HIV cure and remission attempts.

## CD8^+^ T Cells in the Post-ART Era

**Figure 1:**
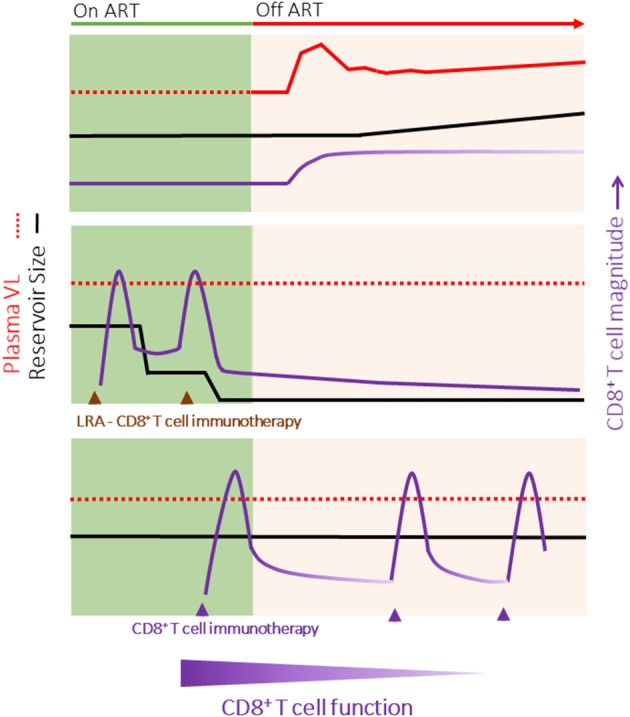
HIV Cure Strategies may require different properties of CD8^+^ T cells. **(Top)** Outline of typical HIV rebound (red line) following the cessation of ART. Although the magnitude of the HIV-specific CD8^+^ T cell response increases, there is a progressive loss of function with time off ART. **(Middle)** HIV Eradication of the replication competent reservoir (black line) combining latency reversal agents and immunotherapies to boost or redirect CD8^+^ T cells (purple line) to rapidly eliminate all cells infected with HIV. Following viral clearance, the magnitude of the HIV-specific CD8^+^ T cell response would decline, but a small population of functional memory cells would persist long-term. **(Bottom)** Durable ART-free remission in which the CD8^+^ T cell host immune response limits HIV rebound without decreasing the size of the HIV reservoir. This strategy may require intermittent boosting of the CD8^+^ T cell response (for example, through immunization) to combat a potential decline in the magnitude and function of HIV-specific CD8^+^ T cell responses over time. It is likely that different functional properties of CD8^+^ T cells will be required for HIV eradication (e.g., rapid killing, penetration of tissue reservoirs) vs. HIV remission (e.g., memory maintenance). Note, HIV eradication and remission strategies may be combined.

The long-term goal of HIV cure is to enable HIV-infected individuals to cease life-long antiretroviral therapy (ART) through the development of strategies to eradicate cells latently infected with HIV. Studies in which patients with little to no measurable HIV reservoir (due to very early ART treatment) rebounded following ART removal suggest that HIV reactivation may originate from a few or even a single replication competent provirus (1, 2). Total eradication of the HIV reservoir, and therefore true HIV cure, while no doubt the ideal, will therefore be challenging to achieve. Consequently, many groups are pursuing strategies to induce durable ART-free remission without HIV rebound. Both *in vivo* and *in vitro* studies support a role for CD8^+^ T cells in HIV eradication and durable remission approaches (3–6). CD8^+^ T cells are highly efficient killers of virus-infected cells; however, HIV-specific CD8^+^ T cells induced by natural infection fail to suppress viral replication after cessation of ART (Figure 1, top), suggesting that a successful HIV cure or durable remission strategy may require the priming of *de novo* HIV-specific responses and/or qualitative shifts in CD8^+^ T cell function. To date, CD8^+^ T cell HIV immunotherapies have been broadly unsuccessful. Failure has been attributed not only to poor population-level immunogenicity but also ongoing immune dysfunction in HIV^+^ART^+^ individuals.

A new generation of HIV therapeutic vaccines have been developed that exhibit greater immunogenicity and efficacy in pre-clinical testing (7, 8). In addition, therapies such as bispecific biologics work by harnessing all CD8^+^ T cells, and therefore promise to be scalable to a large, genetically diverse population (9–11). Success with all of these strategies still however relies on the quality and function of CD8^+^ T cells. Here, we review the global function of CD8^+^ T cells under ART, comparing CD8^+^ T cell characteristics between HIV^+^ART^+^, HIV seronegative individuals (HIV-), and untreated HIV^+^ infected individuals grouped into elite controllers (EC), viremic controllers (VC) and typical progressors (TP). We also summarize literature comparing HIV-specific CD8^+^ T cells in treated and untreated HIV infection.

Overall, CD8^+^ T cells undergo substantial restoration of function following prolonged ART suppression, including in individuals treated in chronic/advanced infection. The phenotype and functional profile of total CD8^+^ T cells in HIV^+^ART^+^ individuals more closely resembles that of HIV seronegative (HIV^−^) than of HIV seropositive (HIV^+^) individuals, including HIV controllers. This supports the continued testing of CD8^+^ T cell immunotherapies for HIV cure. However, CD8^+^ T cells, including HIV-specific CD8^+^ T cells, in HIV^+^ART^+^ individuals resemble the phenotypic and functional profile of CD8^+^ T cells in older HIV^−^ individuals. We postulate that the “immunosenescent” phenotype of CD8^+^ T cells in HIV^+^ART^+^ individuals has differential implications for CD8^+^ T cell immunotherapies targeted at HIV eradication vs. durable remission strategies.

## Total CD8^+^ T Cells Under ART

Untreated HIV infection causes progressive CD8^+^ T cell dysfunction, skewing T cell differentiation and limiting CD8^+^ T cell proliferation, cytokine production and lytic function (12–17). In untreated infection, sustained HIV viremia is a major driver of CD8^+^ T cell dysregulation. In individuals in whom viremia is lower, broader T cell function is observed (14, 18, 19). EC and VC, who typically control viremia in acute/early HIV infection, consistently exhibit a broader range of CD8^+^ T cell cytokine production and higher lytic function than typical progressors (TP) (Table 1). Similarly, CD8^+^ T cells from individuals who initiate ART in early infection mostly exhibit broader function than those treated in chronic infection (16, 64, 65). Early virus control also limits other drivers of CD8^+^ T cell dysfunction, including CD4^+^ depletion and lymph node fibrosis (66–68).

**Table 1:** Total T cell characteristics in HIV seronegative and HIV seropositive participants *relative* to HIV seropositive, durably ART suppressed individuals^a^ (↑, higher relative to ART treated; ↓, lower relative to ART treated; ≈, comparable to ART treated).

***Global* CD8^**+**^ T cell characteristics**		**HIV seronegative**		**HIV seropositive ART naive**			**Ref**
		**Age < 65**	**Age >65**	**Elite controllers^**b**^**	**Viremic controllers^**b**^**	**Typical progressors^**b**^**	
CD4/CD8 ratio		↑	≈	≈/↑	↓	↓	(20–29)
CD8^+^ subsets	Naïve	↑	≈	↑	↑	≈/↓^**^	(30–36)
	Central memory	≈/↓	≈	≈	≈/↓^**^	≈	(22, 37)
	Effector memory	↓/≈	≈	≈	≈	≈/↑^**^	(30, 31, 37)
	TEMRA	≈		≈/↓^**^	≈	≈	(30, 31)
CD8^+^ T cell activation	% CD38^+^ HLA-DR^+^	≈	↓	↑	↑	↑	(30, 32, 38)
	% PD-1 expression	≈		≈	≈	↑	(22, 31, 39–42)
CD8^+^ senescence	Telomere length	↑	≈	↓	↓	↓	(22, 34, 36, 43–46)
	TCR diversity	↑≈	≈			↓	(32, 46–51)
	%CD27^−^CD28^+^	↑≈	≈			↑	(22, 31, 32, 46, 52)
	%CD57^+^	↓	≈	↑		↑	(22, 35, 37, 44, 53–56)
	%CD27^−^CD28^−^	↓≈	≈				(22, 30–32, 34, 57, 58)
CMV	CMV-specific	↓	↓	≈		↓	(22, 35, 59–61)
CD4 %	Peripheral blood	↑/≈		≈	≈/↓^**^	↓	(13, 45, 62, 63)
	Gut mucosa	↑		≈	↓	↓	(13)

a *Includes immunological non-responders in whom VL is suppressed by CD4 T cells do not fully restore. Generally immunological non-responders exhibit more residual CD8 T cell dysregulation*.

b *Elite controllers: viral load/ml < 50, CD4/μl >350; Viremic controllers: viral load/ml 50–2,000, CD4/μl >350; Typical progressors: viral load/ml >2,000, CD4/μl >350; Progressors (AIDS): viral load/ml >2,000, CD4/μl >200*.

** *non-significant changes reported relative to ART suppressed individuals*.

Under ART, low-level viremia (~1 copy/mL plasma) is detectable in most individuals and likely contributes to the long-term detection of HIV-specific CD8^+^ T cells, and the CD8^+^ T cell phenotype and dysfunction observed (69). The other driver/s of residual CD8^+^ T cell dysregulation are increasingly difficult to assess because of the changing demographics of HIV^+^ART^+^ individuals. Age impacts immune function, and because over 50% of HIV^+^ART^+^ individuals in the U.S. are over the age of 50, age is a confounder in studies of CD8^+^ T cell function in HIV^+^ART^+^ individuals (70).

The primary effect of aging on the immune system is the process of immunosenescence. Features of immunosenescence in healthy individuals include low CD4/CD8 T cell ratio, decreased telomere lengths which limits mitosis, and an increase in total, senescent, terminally differentiated and activated CD8^+^ T cells (71–73). This latter phenotype limits CD8^+^ T cell proliferative and functional capacity. Immunosenescence is also associated with co-morbidities such as elevated risk of cardiovascular disease, cancer, fragility and tissue damage resulting from dysregulated inflammation (74). The incidence of these co-morbidities is also increased in HIV^+^ART^+^ individuals (47).

### CD4/CD8 Ratio Under ART

Untreated HIV infection is characterized by an inverted, low CD4/CD8 ratio (75), that results from both ongoing CD4^+^ T cell depletion and the persistent elevation of peripheral CD8^+^ T cells. While ART consistently improves patient CD4/CD8 ratio, irrespective of pre-ART CD4^+^ counts (76), CD8^+^ T cell absolute counts in untreated infection remain relatively stable post-ART (75). The net result is that ART generally fails to fully normalize the CD4/CD8 ratio to levels measured in age-matched HIV seronegative individuals (20–25, 77) (Table 1), and the consistently low CD4/CD8 ratio observed in ART treated individuals is strongly associated with a number of immunological abnormalities (20, 21, 77). Risk of co-morbidities and mortality are further increased in immunological non-responders in whom ART controls virus load but CD4^+^ T cell recovery is poor (78–80).

HCMV seropositivity, which is higher in HIV-infected individuals (>80%) than the broader population (81), has been consistently implicated as a driver of the elevated global CD8^+^ T cell counts observed in HIV-infected individuals both off and on ART (82). HCMV seropositivity also increases with age, and HCMV serostatus is independently associated with elevated non-AIDS co-morbidities (81, 83). HCMV induces a potent CD8^+^ T cell response that increases in magnitude over time, a phenomenon termed “memory inflation” (84), and HCMV-specific CD8^+^ T cell responses can account for as much as 50% of all antigen-specific CD8^+^ T cells in elderly individuals [reviewed in (85)]. A recent study showed that HCMV^+^HIV^+^ individuals had higher absolute CD8^+^ T cell counts than individuals who were either HCMV^+^ or HIV^+^ alone (86), but it is unclear whether these higher levels resulted only from increased frequencies of HCMV-specific CD8^+^ T cells (22, 59). While HCMV-specific CD8^+^ T cell responses do not themselves exhibit functional impairment in HIV^+^ART^+^ individuals (22), HCMV infection may indirectly impact CD8^+^ T cell immunoscenescence through ongoing production of proinflammatory cytokines and by limiting T cell receptor diversity across the broader CD8^+^ T cell population (22, 59).

### CD8^+^ T Cell Subsets Under ART

CD8^+^ T cell differentiation and maturation is skewed during HIV infection (Table 1) (30, 31, 87). Naïve (CD45RA^+^ CD27^+^) CD8^+^ T cells are depleted from early infection, more so in TP than EC and VC (30, 87). ART partially restores naïve CD8^+^ T cells relative to TP, but frequencies are more comparable to those of older HIV- than age-matched healthy individuals. Differences in terminally differentiated (CD45RA^+^CD27^−^), central memory (CD45RA^−^CD27^+^) and effector memory (CD45RA^−^CD27^−^) subsets between ART treated and untreated HIV-infected individuals, although less striking, have also been reported (30, 31, 87). The failure to fully restore the CD8^+^ naïve compartment combined with elevated total CD8^+^ T cell frequencies described above suggests that, similar to observations in older people, induction of *de novo* CD8^+^ T cell responses in HIV^+^ART^+^ individuals may be more limited.

There have been few reports of induction of novel HIV-specific CD8^+^ T cell responses following therapeutic vaccination of ART^+^HIV^+^ individuals (88), though this in part may arise from the limited immunogenicity of first generation T cell vaccines. Whether it will be possible to induce potent *de novo* HIV-specific T cell responses in HIV^+^ART^+^ individuals, equally important for both HIV eradication and remission approaches, is a key question in the current era of CD8^+^ T cell immunotherapy.

### Immune Activation Under ART

Uncontrolled HIV replication is characterized by elevated frequencies of CD4^+^ and CD8^+^ T cells expressing activation markers such as CD38 and HLA-DR (89, 90). The importance of immune activation in HIV pathogenesis is underscored by the observation that in untreated infection, T cell activation predicts disease progression independently of viral load (91–93). In individuals with durably suppressed viral load on ART, peripheral T cell activation is substantially reduced relative to untreated individuals, including EC (30, 32, 38). However, in many cases, T cell activation is not fully normalized relative to HIV-uninfected individuals (22, 94, 95). Residual T cell activation appears to be particularly prevalent in immunologic non-responders (96, 97).

As introduced earlier, low-level viremia is observed in most HIV^+^ART^+^ individuals (98, 99). These levels are lower than observed in EC, who exhibit higher T cell activation (100). Activation levels are higher again in VC and TP (30, 32, 38). Other drivers of elevated T cell activation may be an ongoing consequence of immune dysregulation prior to the initiation of ART. These include lymphoid fibrosis, the depletion of regulatory T cells (Tregs) with anti-inflammatory activities, and loss of gut barrier integrity leading to translocation of bacterial products such as LPS that could trigger inflammatory responses (69, 101–103). This has sparked interest in whether it might be possible to normalize immune activation through early initiation of ART (often defined as ART within 6 months of infection), perhaps by minimizing the size of the viral reservoir and preserving regulatory T cells, the integrity of lymphoid architecture, and the gut epithelial barrier. Research in this area has produced conflicting results, indicating that the effect of early ART on T cell activation may depend on how early after infection ART is initiated, how long after ART initiation activation is measured, and other, as yet undefined, factors (104–108).

Residual T cell activation may have implications for HIV cure. Activated CD4^+^ T cells more readily support productive HIV replication, potentially rendering them more vulnerable to infection in any HIV cure attempt involving analytic treatment interruption (ATI) (109). Activation-induced cell death of uninfected (or abortively infected) “bystander” CD4^+^ T cells could also contribute to CD4^+^ depletion during ART treatment interruption (110, 111). Conversely, activated CD4^+^ T cells harboring replication-competent but latent virus may be more amenable to latency reversal. Notably, some putative latency-reversing agents activate T cells *in vitro* (112–114).

### CD8^+^ T Cell Function in Lymphoid Tissues Under ART

Most research on CD8^+^ T cell function under ART conducted to date has been performed on peripheral blood mononuclear cells (PBMC). While the blood is the easiest compartment to sample, most HIV replication occurs in other tissues, particularly mucosal and lymphoid tissue (115–117). These tissues harbor the bulk of HIV RNA^+^ cells in HIV^+^ART^+^ individuals (118). Polyfunctional HIV-specific tissue resident memory (TRM) CD8^+^ T cells are found at higher frequencies in the gastrointestinal tract of EC compared with individuals on ART, suggesting that in EC these cells may play an important role in *in vivo* viral suppression (119). Current available data indicate that in both HIV^+^ and HIV^−^ individuals, memory CD8^+^ T cells in the lymph nodes and rectal mucosa express less perforin and granzyme B and are less efficient killers of target cells than their counterparts in the peripheral blood (120–122). This less cytotoxic phenotype may be related to the function of lymphoid tissue as predominantly a site of lymphocyte priming and maintenance. Furthermore, *de novo* perforin production is lower in *ex-vivo* stimulated HIV-specific CD8^+^ T cells from EC compared with typical progressors, suggesting that perforin production may not be the major control mechanism in the gut, and that cytokine production in lymphoid tissues may be a more useful correlate of virologic suppression (13, 121).

In secondary lymphoid tissues (LT), HIV replication is concentrated within CD4^+^ T cells in the B cell follicles (123). This may be a consequence of the partial exclusion of HIV-specific CD8^+^ T cells from follicles (123–126). HIV replication is also associated with LT fibrosis, which strongly correlates with the depletion of naïve CD4^+^ T cells, and is inversely correlated with the extent of immune reconstitution upon the initiation of ART (66). LT fibrosis, caused through the deposition of collagen by T regulatory cells, disrupts LT architecture, resulting in T cells with less access to antigen and IL-7, which is critical for T cell maturation and maintenance (66, 127). LT fibrosis is elevated in all HIV^+^ groups (HIV^+^ EC, VC, TP, and HIV^+^ART^+^, both immunologic responders and non- responders), compared to HIV^−^ individuals (128). To date, the data suggest that LT fibrosis does not reverse with ART; however, early initiation of ART may limit the viral replication-dependent inflammation that drives fibrosis, improving immune reconstitution (66, 128). These additional barriers, need to be considered for HIV cure or remission strategies. Strategies to redirect CD8^+^ T cells to immune-privileged sites, such as the follicles, and/or strategies to limit LT fibrosis or reverse collagen deposition, such as IL-7 therapy are being investigated (129, 130).

### CD8^+^ T Cell Senescence Under ART

Telomere integrity is critical for mitotic division and cell survival. The shortening of telomeres is a hallmark of decreased cell proliferation and can activate pathways resulting in apoptosis or cellular senescence (22, 131–133). Compared to HIV^−^ individuals, the telomeres of CD8^+^ T cells in both HIV-infected ART naïve or treated individuals are significantly shorter, indicating a history of increased cell divisions (22, 43, 134). Though multiple studies suggest that T cell telomere length is partially restored (relative to untreated infection) following ART, ongoing proliferation defects in ART treated individuals have been confirmed(Table 1) (112, 135–137). Similar to alterations in T cell differentiation state, telomere length in T cells is more consistent with that of older seronegative individuals (47).

CD8^+^ T cells with shortened telomeres exhibit a combination of CD28^−^, CD57^+^, or CD27^−^ phenotypes (138–141). An increased frequency of CD28^−^CD57^+^ CD8^+^ T cells is observed in HIV infection, where elevated CD57^+^ expression has been associated with reduced proliferative capacity of CD8^+^ T cells (22, 53, 142). Initiation of ART in early infection is able to largely normalize the proportion of CD8^+^ CD28^−^, CD57^+^, and CD27^−^ T cells, though not to the levels seen in age-matched HIV-seronegative individuals (142). Expression of senescence markers in HIV^+^ ART^+^ individuals again resembles that of older seronegative individuals.

A highly diverse T cell repertoire is generally associated with an effective immune system and efficient control of chronic viral infections. HIV infection is associated with qualitative TCR repertoire changes, including disruption of the TCR variable region, Vβ, with CD8^+^ T cells affected to a greater extent than CD4^+^ T cells (143, 144). Compared to HIV^−^ individuals, HIV^+^ treatment-naïve individuals exhibit a significant decrease in whole-blood TCR diversity (145). However, the TCR repertoire diversity of naïve and memory/effector sub-populations is comparable between HIV^+^ and HIV^−^ individuals. This suggests that the decrease in TCR repertoire diversity in the blood results from the expansion of more differentiated T cell populations with lower TCR diversity (145). Most (48, 49, 146), but not all (147), studies have observed that ART is unable to fully reconstitute the TCR repertoire in CD8^+^ T cells to the diversity seen in seronegative individuals. Whether the diminished TCR diversity in HIV^+^ART^+^ individuals will impact curative strategies is unclear as co-factors separate from TCR diversity also contribute to CD8^+^ T cell function (148).

### Summary

Collectively, the current available literature suggests that durable viral suppression under ART is associated with partial normalization of the frequency, activation, differentiation, senescence, and diversity of global CD8^+^ T cells. The overall, global CD8^+^ T cell profile in HIV^+^ART^+^ individuals is more similar to HIV seronegative than HIV seropositive individuals, but when HIV seronegatives are stratified by age, data consistently indicate that CD8^+^ T cells in HIV^+^ART^+^ individuals show an aging phenotype.

Since existing HIV-specific CD8^+^ T cells have, by definition, failed to control viral replication in individuals on ART, a successful HIV cure or remission strategy may require the priming of *de novo* HIV-specific responses by vaccination. Reduced TCR diversity, lower frequencies of circulating naïve CD8^+^ T cells, and accumulation of terminally differentiated and senescent cells could hinder this approach by reducing the pool of CD8^+^ T cells available for priming. Further studies are required to definitively elucidate whether early ART or other interventions could preserve CD8^+^ T cell diversity, stemness, and self-renewal capacity.

## HIV-Specific CD8^+^ T Cells Under ART

HIV infection induces a robust HIV-specific CD8^+^ T cell response. In untreated infection, the HIV-specific CD8^+^ T cell response is highly dynamic. CD8^+^ T cells exert selection pressure on HIV, resulting in the emergence of non-synonymous mutations in and around T cell epitopes (149–151). These mutations can result in HIV-infected cells either failing to present the viral epitope or, if presented evading recognition by CD8^+^ T cells (17, 151–154). This process, termed “escape” is first observed within weeks of transmission and continues through infection (149–151). Virus escape results in an ongoing shift in the HIV-specific CD8^+^ T cell response pattern, with new CD8^+^ T cell responses constantly emerging (154). However, HIV infection is also characterized by a progressive loss of HIV-specific CD8^+^ T cell immune function, specifically the loss of the capacity to simultaneously produce antiviral cytokines and release lytic molecules following antigenic stimulation (14, 16, 16). HIV-specific CD8^+^ T cells also exhibit shorter telomeres and poorer proliferative capacity (32, 137). This increasing loss of function in CD8^+^ T cells is associated with upregulation of immune checkpoint markers such as PD-1, CD160, Tim-3, and TIGIT, typically referred to as an “exhausted” phenotype (39). By contrast, HIV-specific CD8^+^ T cells in EC that largely control HIV viremia early in acute-early infection exhibit broader CD8^+^ T cell function, robust proliferation and lower immune activation (16). Here we consider the magnitude, breadth and functional phenotype of CD8^+^ T cells in ART treated individuals compared to TP, EC and VC.

### HIV-Specific CD8^+^ T Cell Magnitude and Breadth

T cell magnitude can be measured by either the absolute frequency (multimer staining to detect all HIV-specific cells, whether functional or not) or by assessing the frequency of functional cells (ELISpot, intracellular cytokine staining, CSFE proliferation etc). Magnitude is an important measure of T cell potency. In HIV infection, the functional magnitude of T cells, measured by IFN-γ ELISpot, is correlated to the time to viral escape, although associations between the overall frequency of HIV-specific CD8^+^ T cells using functional assays and viral load have not been consistently observed (65, 155, 156). Viremia is a primary driver of the magnitude of the HIV-specific T cell response, an observation underscored by the differences in HIV-specific T cell magnitude between groups of HIV infected individuals, including ART treated individuals (155, 157, 158). In a study examining the total summed magnitude of CD8^+^ T cell responses against the entire HIV proteome from PBMCs, as measured by *ex vivo* IFNγ ELISpot, the summed magnitude of CD8^+^ T cells in untreated chronic infection is at least 2-fold greater than the summed magnitude of CD8^+^ T cells from treated infection (Table 2) (155). EC, who control HIV better than TP and have low viral load, exhibit significantly lower T cell responses than TP. In turn, EC, who exhibit higher viremia than ART^+^HIV^+^, have higher magnitude T cell responses (155, 157, 158). Even so, the HIV-specific T cell response in ART treated individuals remains detectable over time, with responses detectable *ex vivo* after years of durable suppression (155).

**Table 2:** HIV-specific CD8^+^ T cell responses in HIV seropositive, treatment naïve individuals *relative* to HIV seropositive durably ART suppressed individuals. (↑, higher relative to ART treated; ↓, lower relative to ART treated; ≈, comparable to ART treated).

**HIV-specific CD8^**+**^ T cell response**		**Elite controllers^**a**^**	**Viremic controllers^**a**^**	**Typical progressors^**a**^**	
Magnitude		↑	↑	↑	(12, 14, 16, 18, 19, 95, 155, 159–161)
Breadth		↑	↑	↑	(18, 19, 155, 162)
Immunodominant protein		Gag	Gag	Env	(14, 18, 163, 164)
*In vitro* function	Polyfunctionality^b^	↑	↑	↓	(12–16)
	Viral inhibition	↑	↑	↓	(5, 18, 19, 165, 166)
	Proliferation	↑	↑	↓	(13, 18, 32, 40–42, 137, 158, 165, 167–171)
Phenotype	% HLA-DR^+^ CD38^+^	≈	↑	↑	(61, 172)
Exhaustion	% PD-1	≈		↑	(39–41, 165)
	% LAG-3			≈	(39)
	% CD160			↑	(39)
	% 2B4			≈	(39)
	% TIGIT	↓		↑	(39, 173)
Survival factors^c^	Cleaved caspase 3- proapoptotic	↓	↑	↑	(174)
	BCL-2 antiapoptotic	↑	↓	↓	(174)

a *Elite controllers: viral load/ml < 50, CD4/μl >350; Viremic controllers: viral load/ml 50–2,000, CD4/μl >350; Typical progressors: viral load/ml >2,000, CD4/μl >350; Progressors (AIDS): viral load/ml >2,000, CD4/μl >200*.

b *Polyfunctionality: expression of multiple cytokines and chemokines (ex. IFNy, TNFα, IL-2)*.

c *Proapoptotic marker, cleaved caspase-3 (CC3) and antiapototic marker (Bcl-2), regulate the mitochondrial released of cytochrome C to induce apoptosis. Bcl-2 negatively regulates the induction of the apoptotic pathway*.

The magnitude of the total HIV-specific CD8^+^ T cell responses positively correlates with the number of reactive epitopes targeted, that is the breadth of response (155). Similar to magnitude, the breadth of response is decreased during ART treatment, and timing of ART initiation affects the breadth of CD8^+^ T cell response (155). Treatment during acute infection results in breadth of response being significantly lower and more narrowly directed than individuals with untreated or intermittently treated HIV infection (Table 2) (155, 175).

### HIV-Specific CD8^+^ T Cell Targeting of HIV

The specificity of the CD8^+^ T cell response may also be critical in HIV control. Associations between the targeting of HIV-specific CD8^+^ T cell responses and virus set point have been observed. In cohort studies of individuals in South Africa with chronic clade C HIV untreated infection, viral load was inversely correlated with the breadth of Gag-specific T cell responses, and was directly correlated with the breadth of Env-specific responses (163). Similar observations were observed in chronic, HIV-clade B untreated infection, where individuals with lower viral loads more extensively target HIV-Gag (176, 177). Work by Mothe and colleagues extended these previous findings (178). T cell responses against the full HIV proteome were mapped in 950 participants and a set of T cell epitopes were identified as “protective,” based on cohort-level associations with lower viral load (178). The protective epitopes occurred in regions of Gag, Pol, and Vif, while non-protective epitopes that were associated with higher virus loads occurred in regions of Env, Nef, Vpr, and Pol (178).

HIV sequence variability at the population-level can be quantified using a measurement called entropy (179). High entropy epitopes are more variable at the population level, whereas low entropy epitopes are more conserved. The “protective” epitopes described by Mothe et al. typically exhibited lower entropy relative to non-protective epitopes (178). Lower entropy regions are less likely to accommodate escape mutations without inducing a fitness cost (17, 154, 180). For example, Gag is subject to more stringent sequence constraints than Env, making it less likely for HIV to accommodate a mutation in this region. More generally, eliciting CD8^+^ T cell responses against the most highly-conserved regions of HIV may be a good strategy for immunotherapies, as escape mutations in these regions can result in fitness cost for HIV.

Virus escape mutations were identified in HIV provirus over 25 years ago (181). More recently, virus escape was also confirmed in the replication-competent reservoir in durably ART suppressed individuals, highlighting the challenge of pre-existing virus escape to T cell immunotherapy strategies, whether for HIV eradication or durable remission (182). Further studies are needed to quantify the level and extent of pre-existing virus escape in the HIV reservoir.

### HIV-Specific CD8^+^ T Cell Function Under ART

In chronic untreated infection, HIV-specific CD8^+^ T cells progressively lose the capacity to proliferate and secrete cytokines and cytotoxic effectors, though even in late-stage infection some function is retained (183–185) (Table 2). ART significantly normalizes HIV CD8^+^ T cell functions (186). However, multiple studies have reported that HIV-specific CD8^+^ T cells from individuals durably suppressed on ART do not exhibit the same breadth of function as HIV-specific CD8^+^ T cells from EC (40, 165). Relative to EC, CD8^+^ T cells from HIV^+^ART^+^ individuals display reduced proliferative capacity and cytokine and lytic molecule production following stimulation with HIV antigens (13, 137, 158, 165, 167, 170). Even more pertinently, HIV-specific CD8^+^ T cells from ART-suppressed individuals have reduced capacity to eliminate both productively infected and latently infected CD4^+^ T cells compared with CD8^+^ T cells from EC (5, 165, 166). Although these observations were likely to be influenced by increased frequencies of HIV-specific CD8^+^ T cells in EC or stronger targeting of protective, low entropy T cell epitopes (13, 165), together they indicate that HIV-specific CD8 T cells in HIV^+^ART^+^ individuals while broadly functional, are not optimal.

### HIV-Specific CD8^+^ T Cell Exhaustion

In untreated HIV infection, progressive functional impairment of CD8^+^ T cells is accompanied by the upregulation of “immune checkpoint markers” such as PD-1, CD160, 2B4, LAG-3, and TIGIT, (with negligible expression of TIM3 and CTLA-4), which can inhibit signaling downstream of the TCR on HIV-specific CD8^+^ T cells, and in chronic viral infections, promote apoptosis (39, 171, 187, 188). Checkpoint marker upregulation in dysfunctional CD8^+^ T cells is a signature of T cell exhaustion. As exhaustion is partly driven by chronic antigen exposure, it would be expected that viral suppression under ART would be associated with at least partial recovery of HIV-specific CD8^+^ T cell function, and this does appear to be the case for multiple markers when HIV^+^ART^+^ are compared to TP (Table 2)(41, 186, 189). Literature are limited on whether checkpoint inhibitor levels remain elevated relative to the HIV seronegative population; however, Tauriainen et al. report that HIV-specific CD8^+^ TIGIT^hi^ cells were associated with lower function in durably treated participants and co-expressed other exhaustion markers, suggesting some ongoing T cell exhaustion in durably suppressed individuals (173).

Antibody blocking of checkpoint inhibitors, with most focus on the PD-1/PDL-1 pathway, can increase CD8^+^ T cell function and, remarkably, in some cancer patients has afforded complete clinical response (190). Clinical data are more limited in HIV-infected individuals. A small clinical study found evidence that low level anti-PD-1 treatment increase CD8^+^ T cell functionality in a subset of durably ART-suppressed HIV seropositive participants, and more recently CTLA-4 blockade was reported to be well tolerated in HIV-infected individuals (191, 192). In SIV-infected macaques, α-PD-1 antibody given 10 days prior to ART initiation increased antiviral CD8^+^ T cell function and produced more rapid suppression and CD4^+^ T cell reconstitution following ART initiation (193). We anticipate that results from several ongoing clinical and non-human primate studies will be reported over coming years.

### Summary

The initiation of ART, particularly during acute infection, is associated with a decrease in HIV-specific CD8^+^ T cell response magnitude and breadth. A HIV cure or remission strategy may require redirecting CD8^+^ T cells to the more highly conserved regions of HIV, as escape mutations in these regions can result in a fitness cost for the virus. When designing immunotherapies intended to elicit CD8^+^ T cells capable of clearing reactivated latent cells, increasing the frequency and redirecting CD8^+^ T cells may not be enough, and specifically, the implications of sub-optimal HIV-specific CD8^+^ T cell function should be considered.

## Immunotherapy in Cure

### CD8^+^ T Cell Vaccines and Therapies for HIV Cure

In most people, the CD8^+^ T cell response to HIV is inadequate to prevent virus rebound. As detailed above inadequate CD8^+^ T cell magnitude and breadth, pre-existing escape in the HIV reservoir, insufficient restoration of CD8^+^ T cell function following ART and ongoing exclusion of CD8^+^ T cells from virus reservoirs such as B cell follicles likely all contribute to the failure of CD8^+^ T cell immunity to control HIV rebound. However, a recent study observed that dual bNAb treatment in the first weeks of macaque simian/human immunodeficiency virus (SHIV) challenge in rhesus macaques resulted in lower persistent viremia (194). SHIV rebound was observed following CD8-antibody depletion in controller animals. In most animals virus control was regained following restoration of CD8^+^ T cells (194). While this study design cannot be easily translated into clinical practice, the observations provide proof-of-principle that CD8^+^ T cell immunity can be augmented, resulting in improved long-term virus control.

CD8^+^ T cell vaccine therapies against HIV aim to stimulate pre-existing and/or generate *de novo* HIV-specific CD8^+^ T cell responses to suppress viral replication through the clearance of HIV-infected cells. To date, therapeutic vaccine regimens, which range from recombinant DNA and viral vectors to dendritic cell vaccines (195, 196), have only shown a modest effect, and a minimal delay in virus rebound following ATI, which may be due to limited immunogenicity of the vaccine [reviewed in (197)]. The limited efficacy of current therapeutic vaccines may also be due to HIV escape or lack of restoration of CD8^+^ T cell function. However, the newest generation of vaccines involve heterologous vector prime-boost regimens, which have shown enhanced immunity compared to homologous regimens, as well as conserved immunogenic designs which may help to overcome population level immunogenicity and virus escape (7, 198). Adoptive transfer of *in vitro* expanded autologous and allogeneic HIV-specific CD8^+^ T cells is also being pursued (199, 200). This approach has been successful against some cancers (6, 201, 202) and to date, HIV-CD8^+^ T cell therapy has been safe in HIV^+^ART^+^ individuals (203).

Other approaches under active investigation seek to harness bulk (rather than antigen-specific) CD8^+^ T cells for HIV clearance. These approaches include the use of chimeric antigen receptors, Dual-Affinity Re-Targeting protein (DARTS) and T cell receptor (TCR)-targeting system with an anti-CD3 effector function (ImmTAVs) in which CD8^+^ T cells are transduced with either a HIV-specific T cell receptor, a HIV-specific binding antibody, HIV-specific antibodies, or T cell receptors ligated to a CD3 effector molecule (9, 10, 204). Several molecules are in Phase I testing or progressing to Phase I testing.

These approaches could all be combined with approaches to block checkpoint inhibitors and enhance CD8^+^ T cell function of CD8^+^ T cell responses or combined with approaches targeting other arms of the immune response such as bNAb therapy. In combination with antiretroviral drugs, CD8^+^ T cell therapies, bNAbs, blockade of regulatory pathways and the harnessing of other immune cells may offer new therapeutic approaches in a near future.

### Integration of CD8^+^ Therapies to HIV Cure

HIV eradication requires reactivation and clearance of latently infected cells that evade ART because they are long-lived and quiescent (205) and/or undergo homeostatic proliferation without reactivation of the integrated provirus (206). Multiple groups are focusing on developing small molecule or immune-based approaches to reactivate HIV latently infected cells. To date, latency reversing agents have successfully increased cell associated HIV RNA in resting CD4^+^ T cells and induced viral blipping *in vivo* (207–209). However, these studies, supported by *in vitro* work (5), suggest that reactivation is transient, with cells rapidly returning to a state of quiescence, and no change in the size of the HIV reservoir was observed. This suggests that CD8^+^ T cells and or other immune effectors have a limited window to clear reactivated cells. The implication for HIV eradication strategies is that latency reversal and immunotherapy should occur in concert; that is CD8^+^ T cell immunotherapy should produce the “best” CD8^+^ T cell response around the time of maximal latency reversal. For eradication strategies, CD8^+^ T cell clearance in short well-timed bursts may be sufficient (Figure 1, middle).

The desired outcome of HIV cure attempts contrasts with HIV remission strategies that do not seek to eradicate the reservoir but rather control and limit HIV rebound in the long term. Here, T cell based immunotherapy would need to afford sustained CD8^+^ T cell surveillance of stochastic HIV reactivation, perhaps over years (Figure 1, bottom). HIV eradication vs. HIV remission strategies therefore may require qualitatively and quantitatively different CD8^+^ T cell responses.

In conclusion, ART at least partially restores lytic function and virus inhibitory capacity of CD8^+^ T cells. This suggests that therapeutic vaccination can drive expansion of HIV-specific CD8^+^ T cell responses, at least in the short-term (210, 211). These intact functions may be sufficient for HIV eradication approaches that are shorter term and rely more on rapid expansion and lytic function of CD8^+^ T cells. However, accelerated aging phenotype of CD8^+^ T cells could be a greater limitation to durable remission approaches. In this case, ongoing proliferation defects, cellular activation and exhaustion may over time, limit long-term efficacy of immunotherapies. For optimal and sustained efficacy, T cell boosting regimens may need to be incorporated in HIV remission strategies as well as adjunct therapies aimed at reversing or limiting CD8^+^ T cell immunosenescence in the ART suppressed population.

## Author Contributions

JAW, GC, and NG all participated in the conception, drafting, and composition of this manuscript.

### Conflict of Interest Statement

The authors declare that the research was conducted in the absence of any commercial or financial relationships that could be construed as a potential conflict of interest.
